# Severe Whip-Like Cervical Tics as an Indication For Thalamic Deep Brain Stimulation: Report of Two Cases

**DOI:** 10.5334/tohm.1010

**Published:** 2025-05-16

**Authors:** Masamune Tsuji, Kei Yamashiro, Takashi Morishita, Atsushi Hirota, Hitoshi Iida, Yasuhiko Baba, Hiroshi Abe

**Affiliations:** 1 Department of Neurosurgery, Faculty of Medicine, Fukuoka University, Fukuoka, Japan; 2 Department of Psychiatry, Faculty of Medicine, Fukuoka University, Fukuoka, Japan; 3 Department of Neurology, Faculty of Medicine, Fukuoka University, Fukuoka, Japan

**Keywords:** Tourette syndrome, deep brain stimulation, cervical spinal cord injury

## Abstract

**Background:**

Cervical spinal cord injury caused by cervical tics associated with Tourette syndrome (TS) is a recognized complication; however, the role of deep brain stimulation (DBS) in mitigating the risk of such injuries remains unclear.

**Case Report:**

We report two cases of TS with severe cervical tics, both of which responded favorably to DBS. In one case, DBS prevented the progression of cervical spinal cord injury, whereas in the other case, it prevented its onset.

**Discussion:**

Poor control of severe cervical tics is a significant risk factor for cervical spinal cord injury, and early consideration of DBS is recommended.

**Highlights:**

This case report presents two cases in which deep brain stimulation (DBS) was effective for patients with Tourette syndrome exhibiting severe cervical tics. Through this report, we demonstrate the potential effectiveness of DBS as a treatment to reduce the risk of cervical spinal cord injury caused by severe cervical tics.

## Introduction

Tourette syndrome (TS) is a childhood-onset neuropsychiatric disorder characterized by multiple motor and vocal tics that last for at least one year [1]. Tic symptoms usually subside before adulthood, but in some cases, TS can develop into a debilitating condition. Severe cervical tics may lead to spinal cord injury [2,3,4,5,6,7,8]. Epidemiological investigations have shown that patients with TS have a significantly increased likelihood of developing cervical spinal disorders, with cervical motor tics recognized as a potential risk factor contributing to these spinal pathologies [9]. However, the optimal approach for managing the risk of cervical spinal cord injury in patients with TS and severe cervical tics remains uncertain [3,4]. Previous reports have described initial TS symptom control with medication [2,3,4,5,6,7] and botulinum toxin injections [7]. Also, in cases where cervical spinal cord injury is concerned due to severe cervical tics, surgical treatment such as posterior laminoplasty and fixation may be indicated [3,4,5,7,8]. However, it has been noted that when cervical fixation is performed without adequate control of TS symptoms, fixation failure may occur [7,8]. In contrast, deep brain stimulation (DBS) has been reported to be effective for treating the symptoms of TS [10,11,12,13,14]. DBS in TS patients with severe cervical tics is anticipated to reduce the risk of developing cervical spinal cord injury and mitigate the progression of spinal cord injury if already present. To date, very few reports have evaluated the efficacy of DBS in preventing cervical spinal cord injury in TS patients presenting with severe cervical tics, and its potential remains unclear.

In this report, we present two cases of TS in which severe cervical tics were successfully suppressed using DBS. These cases highlight that DBS can serve as an effective treatment for patients with severe cervical tics, particularly in reducing the risk of cervical spinal cord injury.

## Case reports

### Case 1

**Figure 1:**
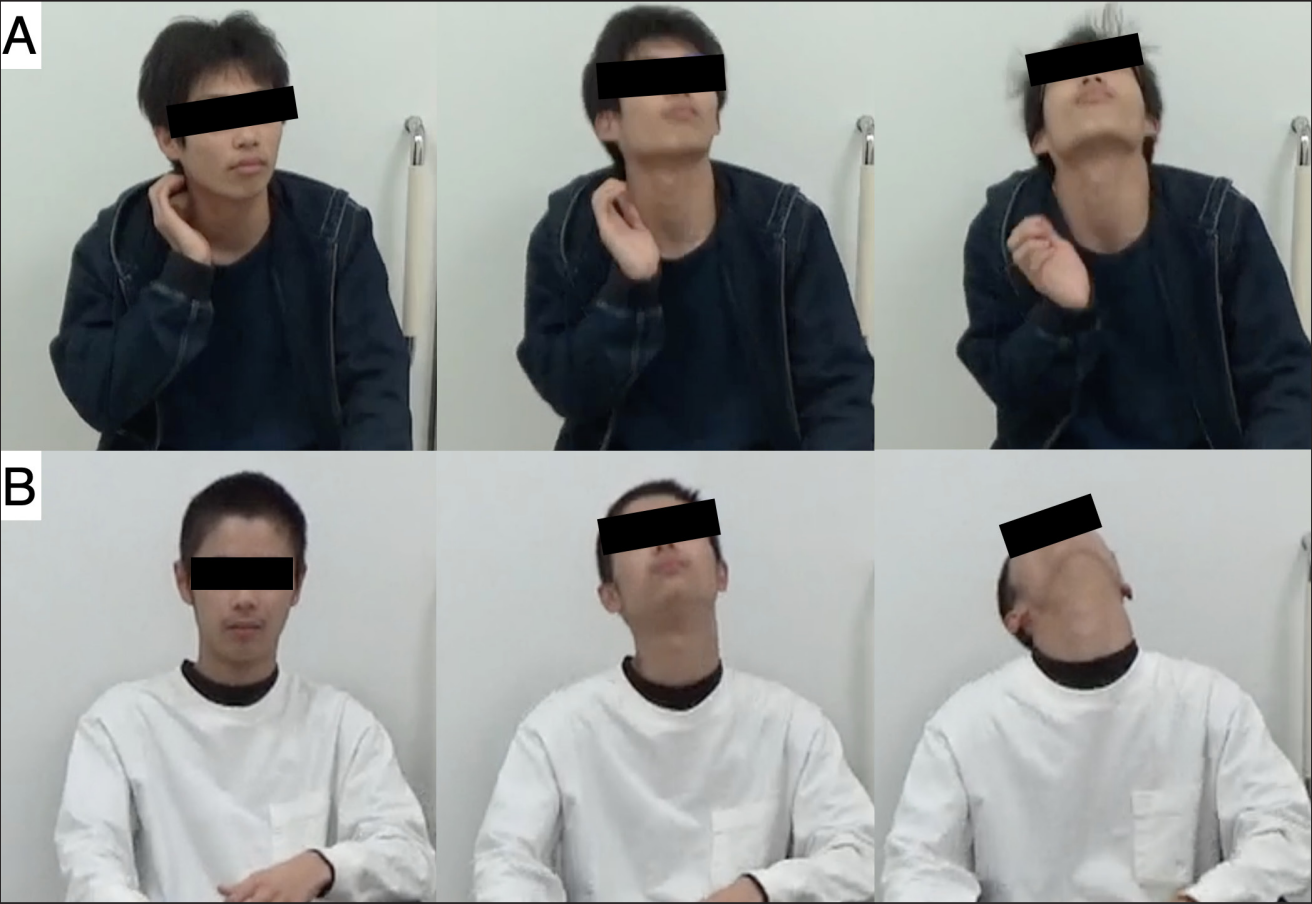
Preoperative sequential photographs of case 1 **(A)** and case 2 **(B)**, demonstrating severe cervical tics with forceful neck extension.

The patient was an 18-year-old male with no abnormalities at birth. Cervical tics appeared when the patient was 5 years old, and he was diagnosed with TS when he was 10 years old. In this case, risperidone, haloperidol, and aripiprazole were trialed. Risperidone and haloperidol did not produce any significant therapeutic effects. In contrast, symptom improvement was observed with aripiprazole, which was continued for four years. The dosage of aripiprazole was adjusted between 3 and 6 mg/day according to symptom severity. Nevertheless, despite continued treatment, adequate symptom control could not be maintained with aripiprazole over time. The patient was referred to our center when he was 18 years old. A preoperative evaluation by a multidisciplinary team (neurosurgery, neurology, and psychiatry) recorded 11.3 tics/min with forceful neck extension (Figure 1A, Video 1), and no comorbidities were identified. On the Yale Global Tic Severity Scale (YGTSS), the severity score was 25 and the impairment score was 50. As the patient was resistant to medical therapy and repeated strong neck extension movements, there were concerns about the risk of cervical spinal cord injury, and DBS was indicated. DBS was performed by targeting the bilateral centromedian thalamic nucleus. We implanted the DBS lead (model 3387; Medtronic, Minnesota, USA) and the implantable pulse generator (IPG) (Activa RC, model 37612; Medtronic).

**Video 1:** Pre- and postoperative videos of case 1 and case 2. They demonstrate motor tics characterized by forceful neck extension and their improvement following deep brain stimulation in both cases. The video has been edited to ensure patient privacy.

A post-DBS evaluation on postoperative day 21 showed symptom improvement, with cervical tics reduced to 4 times/10 min (96.5% reduction). The patient was discharged with a modified Rankin Scale (mRS) score of 1 without any surgical complications. Even at the 5-year follow-up, the effect of DBS continued; the cervical tics had improved to 0 times in 10 min (100% reduction), and the YGTSS severity and impairment scores had improved to 0 (Video 1). The DBS parameters at this time were as follows: left, interleaving stim, 2– (1.0 mA), Case+, 150 microsec, 125 Hz, 3– (1.3 mA), Case+, 180 microsec, 125 Hz; right, interleaving stim, 10– (1.0 mA), Case+, 150 microsec, 125 Hz, 11– (1.3 mA), Case+, 180 microsec, 125 Hz. He is currently employed and living an independent life as a working adult.

### Case 2

The patient was an 18-year-old male with no abnormalities at birth. He developed sniffing tics at age 9 years, followed by vocal and cervical tics at age 10 years, leading to a diagnosis of TS. Obsessive-compulsive behaviors and hypersensitivity symptoms also emerged. In this case, fluvoxamine, aripiprazole, guanfacine, baclofen, and traditional herbal medicines were trialed. Guanfacine, baclofen, and herbal medicines failed to demonstrate efficacy, whereas partial symptom improvement was observed with fluvoxamine and aripiprazole, which were subsequently continued. Aripiprazole treatment was started at the age of 10 and continued at a dose of 2 mg/day, whereas fluvoxamine treatment was initiated at the age of 12 and maintained at 50 mg/day. However, cervical tics, particularly the forceful whip-like neck extension, worsened at 15 years of age.

**Figure 2:**
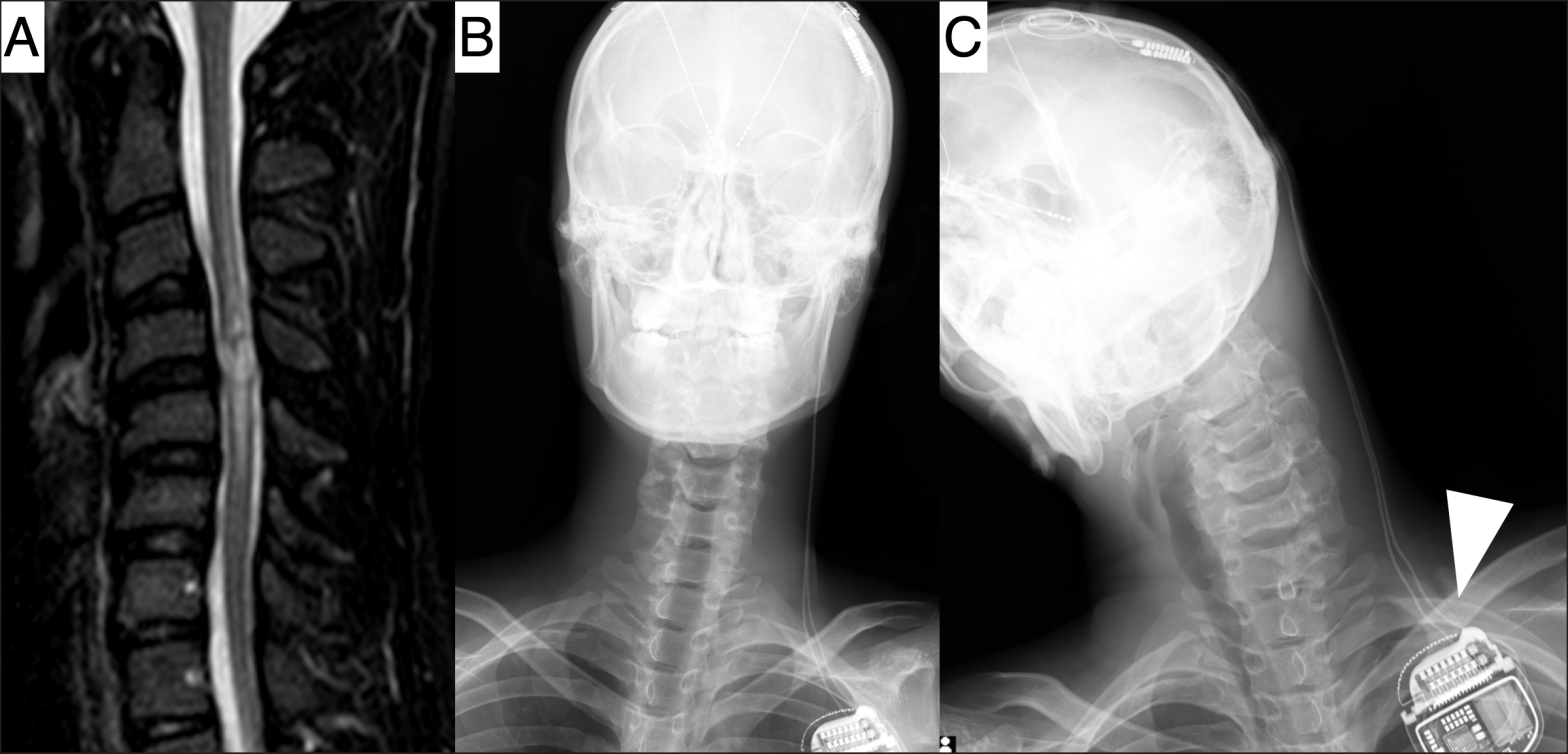
Pre- and postoperative evaluation of case 2. Sagittal T2-weighted magnetic resonance imaging revealed a high-intensity area extending from the C3 to C6 levels **(A)**. Postoperative X-ray confirmed proper lead placement **(B)**. When the neck was extended, the IPG caught on the clavicle, placing stress on the cable (arrowhead), which was presumed to have caused cable breakage **(C)**.

At the age of 17, the patient developed sudden right upper and lower limb weakness. Cervical MRI revealed spinal canal narrowing suggestive of developmental spinal canal stenosis and high-intensity lesions at the C3-C6 levels, consistent with cervical spinal cord injury caused by repetitive cervical tics (Figure 2A). Botulinum toxin injections with ongoing medication failed to control the tics. Cervical fixation surgery was considered but deemed unsuitable because of the risk of fixation failure from uncontrolled tics and psychological distress from hypersensitivity to wearing a cervical collar. Therefore, DBS was indicated as a more aggressive treatment option, and the patient was admitted to our hospital to undergo the procedure.

Pre-DBS evaluation demonstrated quadriparesis predominantly affecting the right side, with manual muscle test scores of 3 in the right and 4 in the left limbs. The deep tendon reflexes were hyperactive in all extremities, and clonus was present in both lower limbs. TS symptoms included frequent vocal and motor tics with forceful neck extension and rapid side-to-side movements, with 19 cervical and 128 vocal tics recorded over 10 minutes (Figure 1B, Video 1). On the YGTSS, the severity score was 42, and the impairment score was 50. The multidisciplinary team (neurosurgery, neurology, and psychiatry) did not identify any spectrum disorder upon evaluation (the autism-spectrum quotient score: 23).

DBS targeting the bilateral centromedian thalamic nucleus was performed. We implanted the DBS lead (model B3301542; Medtronic) and the IPG (Percept RC, model 35300; Medtronic) (Figure 2B). On postoperative day 7, cervical tics reduced to 9 times/10 min (52.6% reduction), vocal tics to 28 times/10 min (78.1% reduction), and the patient was discharged with an mRS score of 3. Manual muscle testing revealed improvement at discharge, with scores of 4 and 5 for the right and left lower limbs, respectively.

No progression of cervical spinal cord injury was observed after DBS surgery. However, at the 3-month follow-up, tics suddenly worsened (cervical: 22 times/10 min, vocal: 105 times/10 min), suggesting a lead or cable fracture. As his major concern was cervical tics, a cable fracture was primarily suspected (Figure 2C).

Two days post-cable replacement, cervical tics improved to 8 times/10 min (57.9% reduction) and vocal tics to 37 times/10 min (71.1% reduction), with an mRS score of 2 at discharge. At the six-month postoperative evaluation, the patient exhibited 10 times/10 min of cervical tics (47.4 % reduction) and 31 times/ 10 min of vocal tics (75.8% reduction), with a YGTSS severity score of 22 and impairment score of 30, indicating sustained efficacy of DBS (Video 1). The DBS parameters at this time were as follows: left, interleaving stim, 1– (1.5 mA), Case+, 90 microsec, 125 Hz, 2– (1.7 mA), Case+, 110 microsec, 125 H; right, interleaving stim, 9– (1.5 mA), Case+, 90 microsec, 125 Hz, 10– (1.7 mA), Case+, 110 microsec, 125 Hz. In addition, no progression of symptoms related to cervical spinal cord injury was observed. The patient currently requires partial assistance with daily activities due to spasticity and paralysis.

## Discussion

DBS is known to be effective in treating TS alongside behavioral therapy, pharmacological treatment, and botulinum toxin injections [10,11,15,16]. Recent guidelines indicate that physicians may consider DBS for severe tics associated with self-injury, including cervical tics, leading to spinal injury [3,4,10]. However, to the best of our knowledge, there are no reports of DBS in patients with cervical spinal cord injury. We chose CM thalamic DBS in our cases, as it represents the most commonly adopted method and no existing guidelines recommend no specific target for TS [17,18].

In case 2, we prioritized tic control through DBS over cervical fixation surgery, achieving both symptom relief and prevention of cervical spinal cord injury progression. In addition, although case 1 initially presented with severe cervical tics, the symptoms completely resolved after DBS. This suggests that the risk of cervical spinal cord injury may have been mitigated. Early consideration of DBS is warranted for TS patients with severe, drug-refractory cervical tics given the risk of cervical spinal cord injury. The mechanisms by which cervical tics lead to cervical spinal cord injury are thought to include direct compression caused by repetitive hyperflexion and hyperextension and traumatic injury to the anterior spinal artery [2,3,4,6]. In these cases, repetitive and forceful neck movements persisted over a long period, and suppressing severe cervical tics was essential to prevent the onset and progression of cervical spinal cord injury. In both cases, no abnormalities other than cervical tics that could have contributed to cervical spinal cord injury were identified. Particularly in patients whose symptoms are insufficiently controlled by pharmacological therapy, appropriate management of cervical tics through DBS may be important for preventing the development or progression of cervical spinal cord injury.

TS complicated by cervical spinal cord injury is rare and an optimal management strategy has yet to be established [3,4]. While there is a consensus that medical treatment should be the first-line approach for symptom control, patients who have undergone long-term medical therapy often have already exhausted various pharmacological options, leaving limited treatment alternatives. In medically refractory patients, cervical fixation surgery has often been performed to prevent the progression of spinal cord injury [3,4,5,7,8]; however, cervical fixation surgery in the presence of persistent cervical tics requires prolonged postoperative use of devices that forcibly immobilize the neck [3,4,5,8]. This imposes a significant physical and psychological burden on patients due to discomfort and restrictions. Moreover, performing fixation surgery without adequate cervical tic control increases the risk of postoperative fixation failure or worsening of symptoms, which may necessitate repeat surgical intervention [7,8]. Indications for cervical fixation surgery in patients with poorly controlled TS remain controversial in clinical practice.

On the other hand, postoperative cable fractures, as observed in case 2, have been reported to occur in 0.7% of patients who undergo DBS surgery, regardless of the underlying condition [19,20]. In addition to cervical dystonia, TS patients are known to sometimes scratch or strike the device, which has been reported to result in a higher frequency of device replacement than in DBS patients with other conditions [10,15]. In case 2, it was suspected that the tension on the cable due to residual neck motion tics after DBS may have contributed to the fracture (Figure 2C). In cases where DBS is effective, sudden symptom exacerbation may indicate cable fractures [20].

Additionally, when performing DBS in patients with TS and severe cervical tics, the risk of cable fractures may be higher than that in typical DBS cases. Thus, providing patients with a thorough preoperative explanation of this risk is crucial, and healthcare providers should remain aware of it. In patients with TS and cervical spinal cord injury, performing cervical fixation surgery for residual motor tics after DBS may reduce the risk of cable fractures and further decrease the risk of cervical spinal cord injury progression. However, defining the appropriate indications and optimal timing of cervical fixation surgery remains challenging. Accumulating similar cases in the future is essential for establishing more precise guidelines.

In conclusion, this case report describes TS patients with violent cervical tics, successfully treated with DBS. Given the risk of cervical spinal cord injury associated with prolonged severe cervical tics, DBS surgery may be indicated in cases where symptoms are not adequately controlled to prevent further spinal cord injury.
